# A GPS Spoofing Generator Using an Open Sourced Vector Tracking-Based Receiver

**DOI:** 10.3390/s19183993

**Published:** 2019-09-16

**Authors:** Qian Meng, Li-Ta Hsu, Bing Xu, Xiapu Luo, Ahmed El-Mowafy

**Affiliations:** 1 Interdisciplinary Division of Aeronautical and Aviation Engineering, The Hong Kong Polytechnic University, Hong Kong; qian2019.meng@polyu.edu.hk (Q.M.); pbing.xu@polyu.edu.hk (B.X.); 2 Department of Computing, The Hong Kong Polytechnic University, Hong Kong; daniel.xiapu.luo@polyu.edu.hk; 3 School of Earth and Planetary Sciences, Curtin University, 6102 Perth, Australia; a.el-mowafy@curtin.edu.au

**Keywords:** spoofing generator, GPS, vector tracking, autonomous vehicles

## Abstract

Spoofing can seriously threaten the use of the Global Positioning System (GPS) in critical applications such as positioning and navigation of autonomous vehicles. Research into spoofing generation will contribute to assessment of the threat of possible spoofing attacks and help in the development of anti-spoofing methods. However, the recent commercial off-the-shelf (COTS) spoofing generators are expensive and the technology implementation is complicated. To address the above problem and promote the GPS safety-critical applications, a spoofing generator using a vector tracking-based software-defined receiver is proposed in this contribution. The spoofing generator aims to modify the raw signals by cancelling the actual signal component and adding the spoofing signal component. The connections between the spreading code and carrier, and the states of the victim receiver are established through vector tracking. The actual signal can be predicted effectively, and the spoofing signal will be generated with the spoofing trajectory at the same time. The experimental test results show that the spoofing attack signal can effectively mislead the victim receiver to the designed trajectory. Neither the tracking channels nor the positioning observations have abnormal changes during this processing period. The recent anti-spoofing methods cannot detect this internal spoofing easily. The proposed spoofing generator can cover all open-sky satellites with a high quality of concealment. With the superiority of programmability and diversity, it is believed that the proposed method based on an open source software-defined receiver has a great value for anti-spoofing research of different GNSS signals.

## 1. Introduction

Autonomous vehicles require an extremely accurate, robust, and reliable navigation system [1,2]. Global Navigation Satellite Systems (GNSSs), such as Global Positioning System (GPS) receivers are heavily relied upon in current autonomous vehicular navigation solutions. However, it is well-known that GPS is vulnerable to interference, such as multipath, jamming, and spoofing [3,4]. The impacts of multipath and jamming can result in a positioning error of several tens of meters or even cause the malfunction of GPS receivers [5,6]. Different from multipath and jamming, spoofing signals are intentionally designed to mislead GPS receivers to fake navigation solutions by generating fabricated synchronized navigation signals [7]. Spoofing seriously limits the use of GPS in applications related to life safety such as autonomous vehicles [8]. Although most GPS receivers have a function to detect and exclude faults, such as receiver autonomous integrity monitoring (RAIM), the need for redundant observations to perform a consistency check still limits its capability in performing anti-spoofing [9,10]. A recent research test on a commercial autopilot system revealed that when facing a spoofing attack implemented by commercially available hardware and software, the vehicle was vulnerable and was spoofed off its intended route easily [11]. This test proved beyond doubt the crucial dependence on GPS for any level 2+ autonomous navigation and the high threat spoofing poses to drivers and passengers utilizing this system.

To generate the spoofing signal, the methods can be broadly divided into meaconing, simulator-based spoofing, and receiver-based spoofing [12]. In meaconing, the GPS signals are recorded and simply replayed after a set delay. This basic meaconing technique, while capable of spoofing encrypted signals, cannot generate an arbitrary trajectory. In simulator-based spoofing, a GPS simulator is used to replicate the signals as they would appear at a chosen location, misleading the receiver to produce an incorrect position, velocity and time (PVT) solution. However, besides the high cost of a commercial signal generator, the software and hardware are not easy to be updated with the development of new signals, channel structures, and navigation message coding rules. In receiver-based spoofing, the receiver processes the actual signals to extract the accurate position and ephemeris. Then the spoofing signals can be generated with the code phase and Doppler shift matching the victim ones at the spoofing position. An advanced receiver-based spoofing technique, which is referred to as nulling, tries to transmit two signals to the victim receiver. One is the spoofing attack signal and the other is the negative of the actual signal. For the signal received by the victim receiver, the actual signal component is cancelled out and only the spoofing component is left. The threat of this spoofing attack is enormous. However, the nulling attack is extremely difficult to be implemented due to exact carrier phase alignment and amplitude matching [13]. In recent research, a way to convert a software-defined receiver (SDR) into a GPS software transceiver was proposed to reuse the sophisticated and optimized infrastructure of the software receiver for the signal generator [14]. This approach makes it possible to realize receiver-based spoofing. The key element in this approach is the usage of software receiver vector-tracking architecture to create the desired line-of-sight (LOS) parameters for updating the numerically controlled oscillator (NCO) and therefore the code and carrier replica generation [15].

Protecting GPS from spoofing is critical to autonomous vehicle navigation and understanding the spoofing mode is the first step to realizing spoofing detection. Spoofing attacks can be divided into two scenarios, an overlapped scenario and a non-overlapped scenario, according to whether the actual signal exists [16]. In a traditional overlapped spoofing scenario, the victim receiver will receive the actual signal and the spoofing signal synchronously. The correlation peak in the tracking channel is overlapped by the spoofing signal and the actual signal. To oppress the actual signal, it is necessary to modify some parameters in the spoofing signal, such as the amplitude and code delay. This kind of spoofing attack is complex and easy to be detected by signal features. Instead, in a non-overlapped scenario, the actual signal is blocked directly, and the victim receiver will receive and process the spoofing signal only. Whether based on communication technology or aided by the urban environment, this scenario is not hard to be implemented. With the recent development of communication technology, the GPS-denied technology can effectively block the actual circumstance. The actual signal will be classified as noise and the spoofing signal will take its place. Particularly, the non-overlapped scenario provides a chance to implement a nulling attack. Compared to GPS-denied technology, the complexity of the urban environment additionally provides many chances to create non-overlapped scenarios in a more natural way. Tall buildings, multi-decked roads, interchanges, and tunnels provide boundaries to block the actual signal. The 3D mapping aided (3DMA) technology can generate both multipath and non-line-of-sight (NLOS) signal interference to facilitate this kind of spoofing [17,18].

Extending from the above spoofing attack on autonomous vehicles, hacker cyberattacks are hazardous and should not be neglected [19], where the non-overlapped scenario still can be created even after the raw signal has been collected by the antenna. The developing hacker cyberattacks make it so that infiltrating the electronic control units and implanting the spoofing signal component are no longer a plot in science fiction or Hollywood movies. The actual signal component will be cancelled and modified to a spoofing signal directly before baseband processing. This internal spoofing solution is more hazardous and concealed compared to external spoofing attacks. The recent anti-spoofing technologies are less able to overwhelm it. 

Many methods have been proposed for spoofing detection, for example, the cryptographic signal method [20,21,22], the multi-sensor aided method [23,24,25], the antenna aided method [26,27,28], and the signal features method [29,30,31]. All these spoofing detection methods show limitations to detection of the non-overlapped spoofing attack, where it can be easily concealed as it does not need to change the signal power or C/N_0_ to suppress the actual signal. The implementation of cryptographic methods is not feasible for civil GPS signals at present. The multi-sensor method is based on the performance of information fusion and the support of various hardware. The aiding sensors also have their limitations in application scenarios, for instance, the vision system cannot work at night. The multi-sensor aided method is not able to work under only receiver available circumstances. The antenna array method is based on more than one antenna and its implementation technology is complicated. In the signal features method, the features of the spoofing signal are quite similar to those of the actual signal and there is no sudden change in the transition process; but still, the signal feature method has not proven to work well. In addition, some crossing methods were proposed to detect spoofing, for instance machine learning [32], maximum likelihood estimation [33], and cooperation of multiple detections [34]. However, these methods are still dependent on prior information or actual signal features [35].

**Figure 1:**
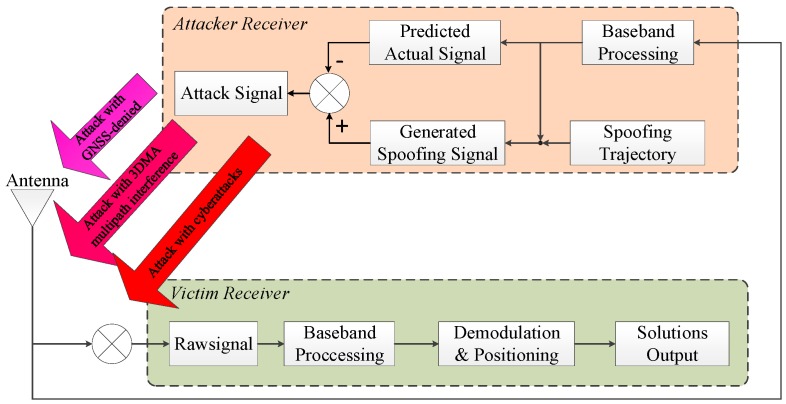
Functional diagram of internal spoofing generator. GNSS is global navigation satellite system; 3DMA is 3D mapping aided.

Furthermore, for a spoofing generator under a non-overlapped scenario, although the actual signal is no longer considered, it is still a key question to connect the actual signal seamlessly at the transition moment. It is easy to detect if out-of-lock happens or if the signal features are different from those of the previous actual signals. On the other hand, creating a vivid spoofing signal almost the same as the actual signal is much more harmful to autonomous vehicles and thus is more helpful to spoofing detection research. In this paper, a GPS spoofing generator based on actual raw signal is proposed. The suggested generator is implemented using the open sourced vector tracking on the SDR platform [36]. Code phase and carrier frequency are generated using a vector delay frequency lock loop (VDFLL) architecture. The proposed spoofing method is suitable for nulling an attack under a non-overlapped scenario. The functional implementation is shown in Figure 1. Firstly, the generator will track the actual signal synchronously to extract the ephemeris of visible satellites, their signal amplitude, and other parameters. Then, the generator will predict the actual signal in the next epoch and generate the cancellation component. At the same time, the spoofing trajectory will be converted to the corresponding spreading code frequency and carrier frequency to generate the spoofing signal component. Finally, the cancellation signal component and spoofing signal component will be combined as the attack signal. The proposed spoofing attack can be launched via a GPS-denied strategy or by using a 3DMA multipath interference approach. In the development of future cyberattacks, the hacker will be able to plant the attack signal into the raw signal. The contributions of this method include two ‘consistency’ and one ‘expansibility’ criteria. The first consistency criterion is that the spoofing signal is generated by modifying the actual signal. The signal power, code phase, and carrier phase are extracted from the actual tracking outputs. The signal features keep consistency with the actual signal. The second consistency criterion is that the proposed method is based on a vector tracking receiver. It can take advantage of the relationship between loop information and receiver states to attack visible satellites to preserve observation consistency. The ‘expansibility’ criterion refers to the detailed implementation based on an open sourced receiver being given. In general, the method is easy to implement and extend to different kinds of satellite navigation systems and signal structures.

The rest of the paper is organized as follows: The design of vector tracking is introduced in Section 2. After that, details about the actual signal prediction and spoofing signal generation based on actual raw signal are given in Section 3. Next, in Section 4, the experimental test evaluating the performance of the proposed spoofing method and the hidden characteristic of the proposed method is analyzed. Finally, Section 5 draws the conclusion. 

## 2. Spoofing Attack Using Vector Tracking 

Vector-tracking is an advanced signal tracking technology, different from the traditional signal tracking, in which all tracking channels are independent to each other and no information exchange is performed between signal tracking. The channels in a vector-tracking receiver are coupled together through the navigation processor. The vector-tracking shows superiority in performance under harsh environments, e.g., increased capabilities against weak signal or high dynamic conditions. In recent years, with the increasing development of intelligent transportation systems and location-based services in urban canyon areas, vector-tracking shows more potential superiorities. For example, vector-tracking is applied to multipath or NLOS reception mitigation in the signal processing stage [37,38]. The fundamental principle behind vector-tracking is the relationship between the code or carrier phase and the receiver states of position, velocity, and time. It gives a feasible opportunity to generate spoofing signals with the given receiver trajectory, as suggested in [14].

**Figure 2:**
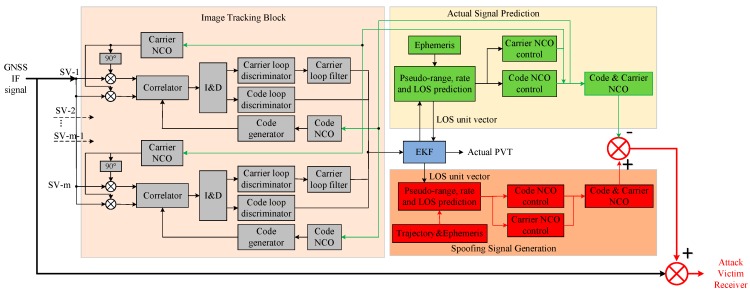
Implementation architecture of the proposed spoofing generator based on vector tracking. ‘SV-m’ represents the m-th satellite. ‘I & D’ means the In-phase and quadrature tracking branches.

In this paper, we use vector-tracking architecture to implement the spoofing attack. From the aspect of demodulating the actual signals, the vector-tracking SDR can track the actual code and carrier much more accurate and robust in urban environments. From the aspect of modulating the spoofing signal, the vector-tracking has the function of converting the predicted receiver position and velocity to the corresponding code frequency and carrier frequency. The detailed implementation architecture is shown in Figure 2. It includes three blocks: tracking channel, actual signal prediction, and spoofing signal generation. All these three blocks are connected with an extended Kalman filter (EKF).

The EKF estimates the actual PVT based on its system propagation and the measurements. After obtaining the navigation solution, the pseudo-range and its rate and the line-of-sight (LOS) vector between the receiver and the satellites are predicted. To do this, the satellite ephemeris data must be known a priori, which means the attacker should process the actual signal and decode the ephemeris data first. The state vector of the EKF is:

(1) $$X={\left[\Delta p_{x},\Delta p_{y},\Delta p_{z},\Delta v_{x},\Delta v_{y},\Delta v_{z},\Delta b,\Delta d\right]}^{T}$$

where $\left[\Delta p_{x},\Delta p_{y},\Delta p_{z}\right]$ and $\left[\Delta v_{x},\Delta v_{y},\Delta v_{z}\right]$ are the three-dimensional receiver position and velocity error vectors in an earth-centered and earth-fixed (ECEF) frame; Δb and Δd are the receiver clock bias and drift in the units of m and m/s, respectively. The system propagation at epoch k is:

(2) $${\hat{X}}_{k}^{-}=\Phi_{k-1}{\hat{X}}_{k-1}^{+}$$

where

(3) $$\Phi_{k-1}={\left[\begin{array}{ccc} I_{3\times 3} & \tau I_{3\times 3} & 0_{3\times 2}\\ 0_{3\times 3} & I_{3\times 3} & 0_{3\times 2}\\ 0_{2\times 3} & 0_{2\times 3} & K \end{array}\right]}_{8\times 8}$$

(4) $$K=\left[\begin{array}{cc} 1 & \tau \\ 0 & 1 \end{array}\right].$$

In Equation (2), τ is the update interval of the EKF. The superscripts “–” and “+” denote the system state before and after measurement update, respectively. The symbol “ ˆ ” represents the EKF estimates. $I_{m\times n}$ represents the identity matrix of (m×n).

The measurement vector can be expressed as

(5) $$Z=\left[\Delta \rho^{j},\Delta {\dot{\rho }}^{j}\right]$$

where $\Delta \rho^{j}$ and $\Delta {\dot{\rho }}^{j}$ are the pseudo-range error and pseudo-range rate error of satellite j, respectively. The detailed calculation method will be given in the following section.

The relationship between the state vector and the measurement vector at epoch k is linearized by a first-order Taylor’s expression as follows:

(6) $$Z_{k}=H_{k}\cdot X_{k}$$

where H is the measurement matrix, calculated as

(7) $$H=\left[\begin{array}{cccccccc} -l_{x}^{1} & -l_{y}^{1} & -l_{z}^{1} & 0 & 0 & 0 & 1 & 0\\ -l_{x}^{2} & -l_{y}^{2} & -l_{z}^{2} & 0 & 0 & 0 & 1 & 0\\ \vdots & \vdots & \vdots & \vdots & \vdots & \vdots & \vdots & \vdots \\ -l_{x}^{m} & -l_{y}^{m} & -l_{z}^{m} & 0 & 0 & 0 & 1 & 0\\ 0 & 0 & 0 & -l_{x}^{1} & -l_{y}^{1} & -l_{z}^{1} & 0 & 1\\ 0 & 0 & 0 & -l_{x}^{2} & -l_{y}^{2} & -l_{z}^{2} & 0 & 1\\ \vdots & \vdots & \vdots & \vdots & \vdots & \vdots & \vdots & \vdots \\ 0 & 0 & 0 & -l_{x}^{m} & -l_{y}^{m} & -l_{z}^{m} & 0 & 1 \end{array}\right]$$

where *m* is the number of satellites involving positioning; the subscript of the LOS unit vector denotes its x, y, and z components, and the superscript denotes the satellite.

The process noise comes from two sources, the receiver dynamics and clock noise, as follows:

(8) $$Q=\left[\begin{array}{cc} Q_{dyn} & 0_{6\times 2}\\ 0_{2\times 6} & Q_{clk} \end{array}\right].$$

The values of $Q_{dyn}$ and $Q_{clk}$ can be set empirically according to the expected receiver motion state and the oscillator used. Alternatively, they can be calculated as

(9) $$Q_{dyn}=\left[\begin{array}{cc} \tau^{3}/3\cdot I_{3\times 3} & \tau^{2}/2\cdot I_{3\times 3}\\ \tau^{2}/2\cdot I_{3\times 3} & \tau \cdot I_{3\times 3} \end{array}\right]\cdot S_{v}$$

(10) $$Q_{clk}=\left[\begin{array}{cc} S_{f}\cdot \tau +S_{g}\tau^{3}/3 & S_{g}\tau^{2}/2\\ S_{g}\tau^{2}/2 & S_{g}\cdot \tau \end{array}\right]$$

where $S_{v}$ is the receiver velocity noise power spectral density (PSD); $S_{f}$ and $S_{g}$ are the PSD of receiver clock phase and frequency, respectively. The value of $S_{v}$ should be set according to the expected level of dynamics. Settings of $S_{f}$ and $S_{g}$ are usually based on the rule of thumb values of the type of oscillator used, or calculated using the following formulas:

(11) $$S_{f}=c^{2}\cdot \frac{h_{0}}{2}$$

(12) $$S_{g}=c^{2}\cdot 2\pi^{2}\cdot h_{-2}$$

where $h_{0}$ and $h_{-2}$ are the coefficients of white frequency modulation noise and flicker frequency modulation noise of the oscillator used, respectively.

The measurement noise covariance matrix is calculated adaptively using the innovation-based adaptive estimation technique. The measurement innovation at epoch k in this paper is

(13) $$V_{k}=Z_{k}-Z_{k}^{-}$$

(14) $$Z_{k}^{-}=H_{k}{\hat{X}}_{k}^{-}.$$

The diagonal element of the measurement covariance matrix is the variance of the measurement innovation. The off-diagonal terms are assumed to be zero due to the weak correlation between channels.

## 3. Actual Signal Prediction and Spoofing Signal Generation

The implementation details of the EKF used in this GPS signal generator are described above. This section will take the advantage of vector tracking to control the local code and carrier generation in two different scenarios: actual signal prediction and spoofing signal generation. Then, the final attacking signal is given after that.

In actual signal prediction, the code NCO control algorithm is implemented using the estimated navigation solution as:

(15) $${\tilde{f}}_{code,k+1}^{j}=f_{CA}\left[1-\frac{{\tilde{\rho }}_{k+1}^{j}-{\hat{\rho }}_{k}^{j}}{c\tau }\right]$$

where ${\tilde{\rho }}_{k+1}^{j}$ and ${\hat{\rho }}_{k}^{j}$ are the predicted pseudo-range at epoch k+1 and the estimated pseudo-range at epoch k; $f_{CA}$ is the code chipping rate (e.g., 1.023 MHz for GPS L1 C/A); c denotes the speed of light. The predicted pseudo-range is calculated using

(16) $${\tilde{\rho }}_{k+1}^{j}=\left\|{\tilde{r}}_{u,k+1}-r_{k+1}^{j}\right\|+\delta {\hat{\rho }}_{sv,c}^{j}+\delta {\hat{\rho }}_{I}^{j}+\delta {\hat{\rho }}_{T}^{j}-{\hat{b}}_{clk}.$$

It consists of two parts: the first part is the predicted range between satellite and receiver, where $r_{k+1}^{j}$ is the satellite position at epoch k+1, which is calculated based on the broadcast ephemeris. ${\tilde{r}}_{u,k+1}$ is the predicted receiver position, which can be calculated based on the system propagation according to Equation (2). The second part is the pseudo-range errors, including the satellite clock error $\delta {\hat{\rho }}_{sv,c}^{j}$, ionospheric delay $\delta {\hat{\rho }}_{I}^{j}$, tropospheric delay $\delta {\hat{\rho }}_{T}^{j}$, and the estimated receiver clock bias ${\hat{b}}_{clk}$, respectively. The receiver clock is also obtained from the propagated EKF state vector.

$f_{code,k+1}^{j}$ is then fed back to the code NCO in each channel to generate local code replicas to keep tracking the actual signal. 

The carrier NCO control algorithm is implemented using the predicted pseudo-range rate at epoch k+1 as follows:

(17) $${\tilde{f}}_{doppler,k+1}^{j}=-{\tilde{\dot{\rho }}}_{k+1}^{j}\frac{f_{L1}}{c}$$

where $f_{L1}$ is the carrier frequency (1575.42 MHz for GPS L1). The predicted pseudo-range rate is calculated using

(18) $${\tilde{\dot{\rho }}}_{k+1}^{j}=\left(v_{sv,k+1}^{j}-{\tilde{v}}_{u,k+1}\right)l^{j}+{\hat{d}}_{u,clk}-d_{sv,clk}^{j}$$

where ${\tilde{v}}_{u,k+1}$ and $v_{sv,k+1}^{j}$ are the velocity vectors of the receiver and satellite *j*, respectively, at epoch k+1; $l^{j}$ is the LOS unit vector from the receiver to satellite *j*; ${\hat{d}}_{u,clk}$ and $d_{sv,clk}^{j}$ are the estimated receiver clock drift and the *j*^th^ satellite clock drift, respectively, both in m/s.

Then, the measurement vector of EKF at epoch k+1 can be obtained from

(19) $$\Delta \rho^{j}=\Delta \tau^{j}\cdot \frac{c}{f_{CA}}$$

(20) $$\Delta {\dot{\rho }}_{k+1}^{j}=f_{doppler}^{j}\frac{c}{f_{L1}}-\left(v_{sv,k+1}^{j}-{\tilde{v}}_{u,k+1}\right)l^{j}-{\hat{d}}_{u,clk}+d_{sv,clk}^{j}$$

where $\Delta \tau^{j}$ is the code discriminator output in chips, $f_{Doppler}^{j}$ is the Doppler shift frequency in Hz.

The mechanism of spoofing code generation is similar to that of actual code prediction. The main difference is that the ‘receiver position’ and ‘receiver velocity’ are replaced by the spoofing trajectory. The spoofing pseudo-range and pseudo-range rates are calculated as:

(21) $${\tilde{\rho }}_{spoof,k+1}^{j}=\left\|r_{trj,k+1}-r_{k+1}^{j}\right\|+\delta {\hat{\rho }}_{sv,c}^{j}+\delta {\hat{\rho }}_{I}^{j}+\delta {\hat{\rho }}_{T}^{j}-{\hat{b}}_{clk}$$

(22) $${\tilde{\dot{\rho }}}_{spoof,k+1}^{j}=\left(v_{sv,k+1}^{j}-v_{trj,k+1}\right)l^{j}+{\hat{d}}_{u,clk}-d_{sv,clk}^{j}$$

where $r_{trj,k+1}$ and $v_{trj,k+1}$ are the spoofing receiver position and velocity extracted from the spoofing trajectory. The details can be found in [14], which includes a 4th degree spline interpolation and a second extrapolation.

### Attack Signal Generation

To generate a whole GPS signal, besides the code and carrier, the amplitude and navigation data are also essential. In the actual signal prediction, the navigation data is obtained from the prompt branch as

(23) $${\hat{D}}_{nav,actual}^{j}=r_{IF}.*C_{prompt}^{j}.*Carr_{\cos}^{j}$$

where $r_{IF}$ is the raw signal, $C_{prompt}^{j}$ and $Carr_{\cos}^{j}$ are the code and carrier in the prompt branch of the satellite j channel. Using ${\hat{D}}_{nav,actual}^{j}$ to generate the actual signal is better as it includes the Doppler residual between two successive epochs. 

In spoofing signal generation, as we do not to consider the Doppler residual, the navigation data is calculated as

(24) $${\hat{D}}_{nav,spoof}^{j}=\{\begin{array}{ll} 1, & if\;I_{p}>0\\ -1, & if\;I_{p}<0 \end{array}\;\mathrm{where}\;I_{p}=\sum_{i=1}^{N_{sample}}\left(r_{IF}.*C_{prompt}^{j}.*Carr_{\cos}^{j}\right)$$

where $N_{sample}$ represents the number of samples in one tracking epoch. 

Regarding the signal amplitude, a simple method to estimate it, as mentioned in [39], is

(25) $${\hat{A}}^{j}=\frac{\sum_{1}^{N_{sample}}\left(r_{IF}.*C_{prompt}^{j}.*{\hat{D}}_{nav,actual}^{j}.*Carr_{\cos}^{j}\right)}{\sum_{1}^{N_{sample}}{\left(C_{prompt}^{j}.*{\hat{D}}_{nav,actual}^{j}.*Carr_{\cos}^{j}\right)}^{2}}.$$

Finally, the attack signal is combined with the predicted actual signal component to generate the spoof signal component as

(26) $$r_{attack}=r_{spoof}-r_{actual}.$$

## 4. Experimental Test and Analysis

**Figure 3:**
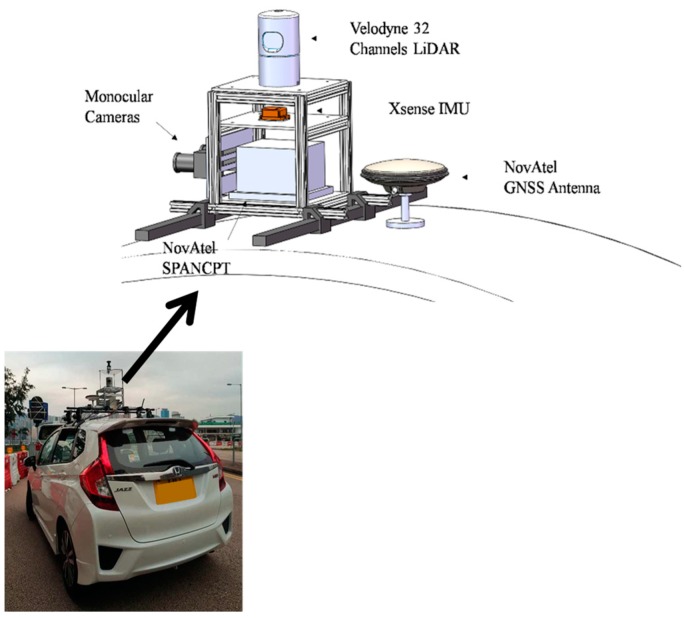
Experimental vehicle platform.

**Figure 4:**
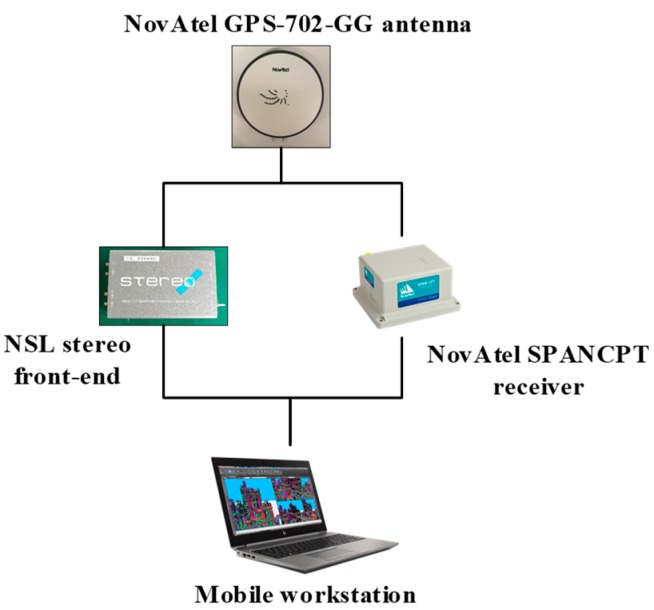
Hardware for signal collect and processing.

Experimental tests were conducted to evaluate the performance of the proposed spoofing generator. The actual signal was collected in a field experiment in Hong Kong and the experimental vehicle platform is shown in Figure 3. The antenna was mounted on the roof of the vehicle. The hardware related to signal collection and processing are shown in Figure 4. NovAtel SPANCPT was used to provide a reference trajectory. GPS signals were collected using a Nottingham Scientific Ltd. (NSL) stereo front-end for post-processing on a mobile workstation. The sampling frequency and intermediate frequency (IF) of the front-end are 26 MHz and 6.5 MHz, respectively. The victim receiver processed the signal with a traditional tracking architecture and least squared positioning mode. The trajectory design, spoofing signal performance in positioning and channel tracking at the transition moment, and the spoofing detection results are analyzed in the following subsections.

**Figure 5:**
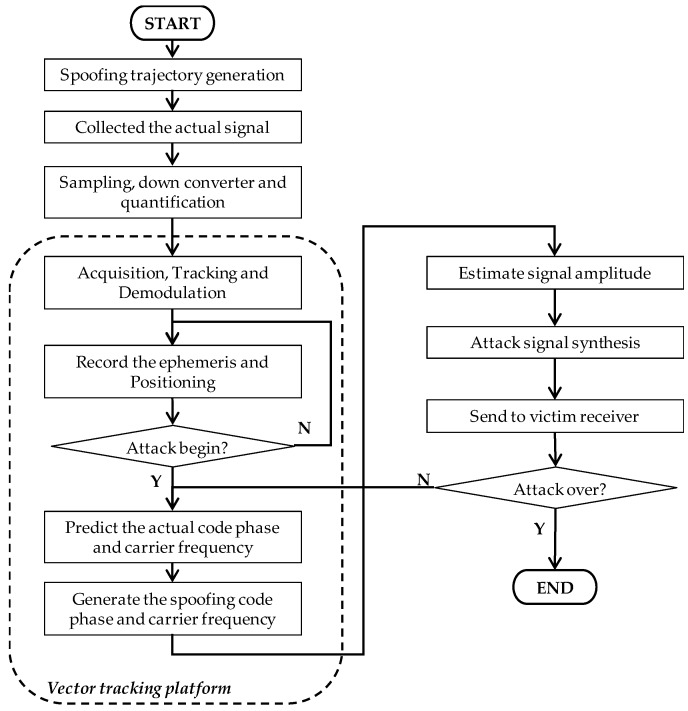
Flowchart of the spoofing generator based on vector tracking.

The proposed method is implemented on the SDR platform with a vector tracking architecture developed by the Positioning and Navigation Lab, Interdisciplinary Division of Aeronautical and Aviation Engineering (AAE), Hong Kong Polytechnic University [36]. The MATLAB software and the corresponding vector tracking open source codes can be downloaded on the GPS Toolbox website [40]. The modular procedure flowchart of the proposed generator execution is show in Figure 5.

### 4.1. Trajectory Design

**Figure 6:**
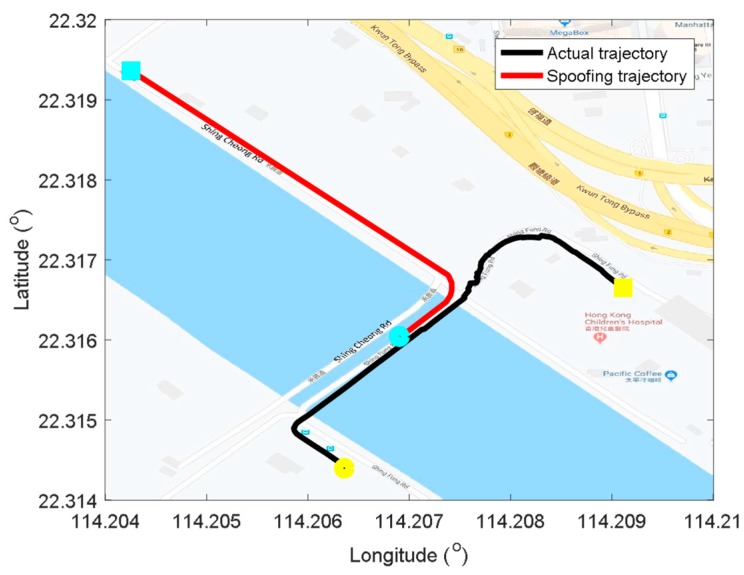
Sketch map for kinematic automobile trajectory. The black and red line are actual and spoofing trajectories, respectively. The starting/terminal points are shown in circle/square points of yellow and blue, respectively.

The detailed test trajectory is shown in Figure 6. The actual kinematic automobile signal was collected along the Shing Fung Road near the Kai Tak Cruise Terminal, Hong Kong. The black line is the actual trajectory. It started from the Kai Tak Cruise side, then crossed the bridge and turned to the southeast. Finally, the experiment terminated near the Hong Kong Children’s Hospital. The vehicle kept static for about 30 s before moving with a moderate speed along the coast. The whole period was about 115 s, including 115,000 positioning epochs. 

The spoofing trajectory was designed on the Google map and also plotted in the same figure as the red line. It is better to use actual roads to generate the spoofing trajectory to meet the physical road constraints of the navigation map in autonomous vehicles. It is easy to connect the spoofing trajectory with the actual trajectory at intersections. As shown in the figure, the spoofing attack was launched from the end of the bridge and aimed to guide the automobile to the Shing Cheong Road, which is parallel to the actual test road but turn to northwest at the end of the bridge. The spoofing attack was launched from the 70^th^ second.

### 4.2. Performance in Positioning 

**Figure 7:**
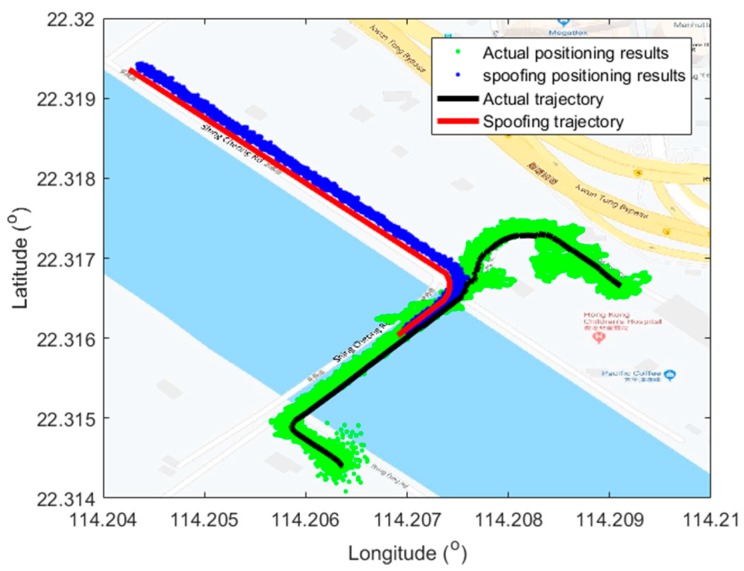
Positioning results plotted in Google map. The green points, blue points, black line, and red line are positioning results under actual signal, positioning results under spoofing signal, the actual trajectory, and spoofing trajectory, respectively.

The act and purpose of spoofing is not only to affect the victim receiver to output the wrong positioning solutions, but also to mislead the receiver to the spoofing trajectory. Actually, the hazard of this type of spoofing attack is much more serious compared to those of the conventional overlapped spoofing attack. The positioning outputs before and after the spoofing attack are shown in Figure 7, also plotted on a Google map. 

**Figure 8:**
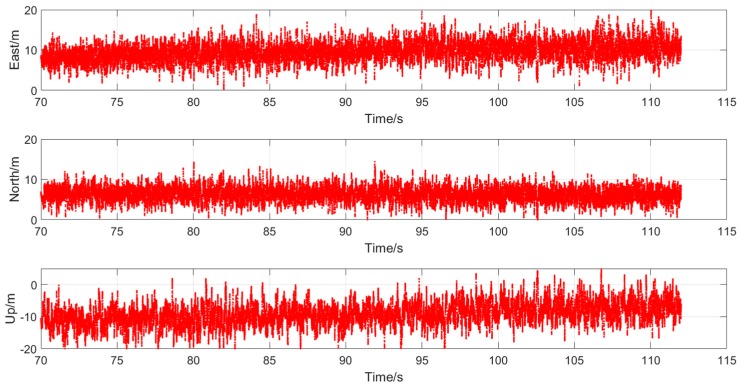
Positioning errors under spoofing attack. From top to bottom: positioning errors in east, north, and up component, respectively.

It is within expectations that the victim receiver was spoofed off its actual trajectory successfully and turned to the Shing Cheong Road at the end of bridge. Then, it kept on working with the established trajectory. What needs to be explained is that the positioning errors under the actual signal in the last half part became bigger due to the interference caused by buildings around the hospital, while the positioning errors under the spoofing signal were small and stable thanks to a relatively open sky along the coast. It is also worth remembering that the spoofing signal generation should consider the impact of surrounding buildings to keep its fidelity, which is considered in our future work. The positioning errors related to the spoofing trajectory are also given in Figure 8, which are given in East–North–Up (ENU) coordinates. The positioning errors are defined as the differences of positioning results and the spoofing trajectory.

As shown in the Figure 7, the values of errors in the three position components kept relatively stable during the whole attack period. This verified the pseudo-range consistency of the whole visible satellites. The superiority of the proposed method was fully shown as the spoofing could cover the visible satellites. Compared to that of the up component, the positioning results in the east and north components matched the spoofing trajectory a little better. This is expected as the positioning accuracy in the horizontal direction is usually better than the vertical direction. Nevertheless, one should note that in positioning and navigation of autonomous vehicles, the horizontal results are of more interest. 

### 4.3. Performance in Channel Tracking 

**Figure 9:**
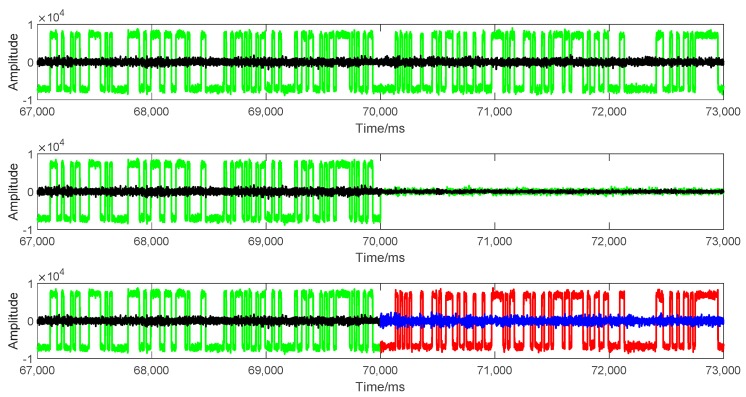
In-phase branch (I_p_) and quadrature branch (Q_p_) outputs of PRN-10 tracking in three different scenarios of signal tracking. The y-axis is the amplitude of coherent integration in 1 millisecond. From top to bottom: (top) when no attacks exist, (middle) actual signal cancelled, and (bottom) actual signal cancelled and spoofing signal modulated. Green and block points represent the I_p_ and Q_p_ outputs of actual signal, respectively. Red and blue points represent the I_p_ and Q_p_ outputs of spoofing signal, respectively.

**Figure 10:**
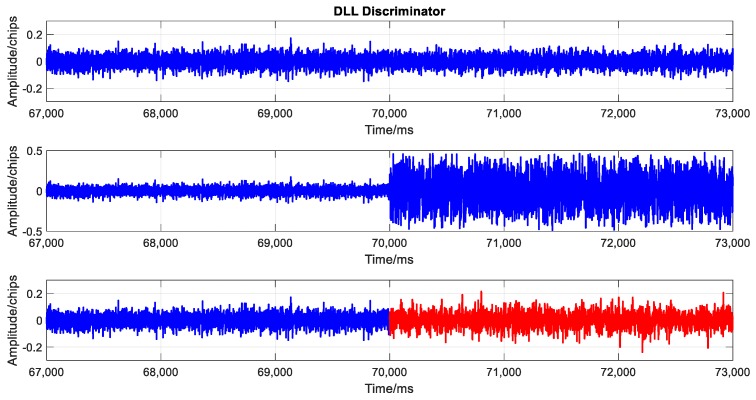
Delay lock loop (DLL) discriminator in the three scenarios. From top to bottom: (top) when no attacks exist, (middle) actual signal cancelled, and (bottom) actual signal cancelled and spoofing signal modulated. Blue and red points represent the outputs of actual and spoofing signal, respectively.

**Figure 11:**
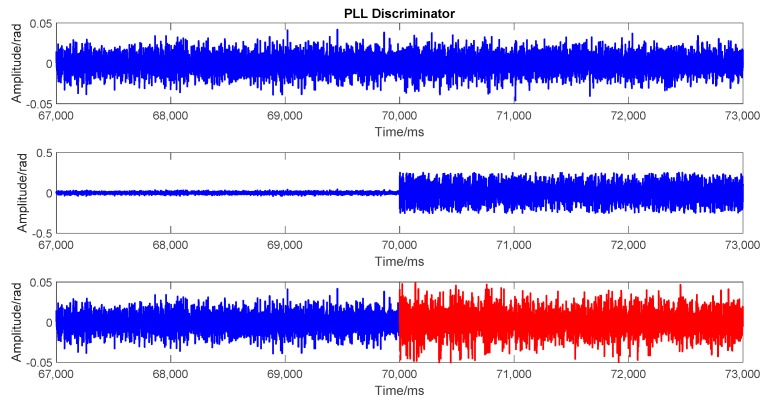
Phase lock loop (PLL discriminator in the three scenarios. From top to bottom: (top) when no attacks exist, (middle) actual signal cancelled, and (bottom) actual signal cancelled and spoofing signal modulated. Blue and red points represent the outputs of actual and spoofing signal, respectively.

To evaluate the performance of spoofing signal further, the tracking results at the transition moment are analyzed in this subsection. Three scenarios are considered in this analysis: (1) actual signal tracking, in which no attack exists; (2) attack with only actual signal cancellation, in which the attack signal only includes the predicted actual signal component; (3) attack with spoofing signal modulated, in which the attack signal not only includes the predicted actual component, but is also combined with the generated spoofing signal component. The tracking results lasted 6 s, including 3 s before spoofing and 3 s after spoofing. The transition point was the 70^th^ second. Figure 9, Figure 10, and Figure 11, respectively, show the outputs of prompt branch, delay lock loop (DLL) discriminator, and phase lock loop (PLL) discriminator in tracking. In every figure, the above three scenarios are presented from top to bottom. Particularly, in the 3^rd^ scenario, the results before and after spoofing are plotted in different colors.

The 2nd scenario shows the results after the actual signal was cancelled. Both the code loop and carrier loop lost lock immediately. There were only noises in the correlations of in-phase branch (I_p_) and quadrature (Q_p_) branch. The actual signal was demodulated and cancelled ideally. A good non-overlapped spoofing attack can be launched in this scenario.

Meanwhile, the tracking results of the 3^rd^ scenario had no obvious difference compared with those of the 1^st^ scenario. There was no outlier or out of lock in the code loop or carrier loop seen from Figure 10 and Figure 11. The amplitude of the correlation outputs of the prompt branch had no significant change from the actual signal to the attack signal, which means that the signal power kept stable at the transition moment. 

### 4.4. Hidden Characteristic for Spoofing Detection 

It seems that the hidden function is the most important characteristic for spoofing attack, especially at the transition moment. The above positioning and tracking results are encouraging from this aspect as there is no abnormal change in the tracking channel after the raw signal are attacked. All the changes at the transition moment are within the receiver normal limits. The victim receiver after spoofing attack can be positioned normally with the spoofing trajectory. The anti-spoofing scheme will not be triggered in this non-overlapped scenario. The machine learning methods would not available as there is no classical spoofing features for training.

**Figure 12:**
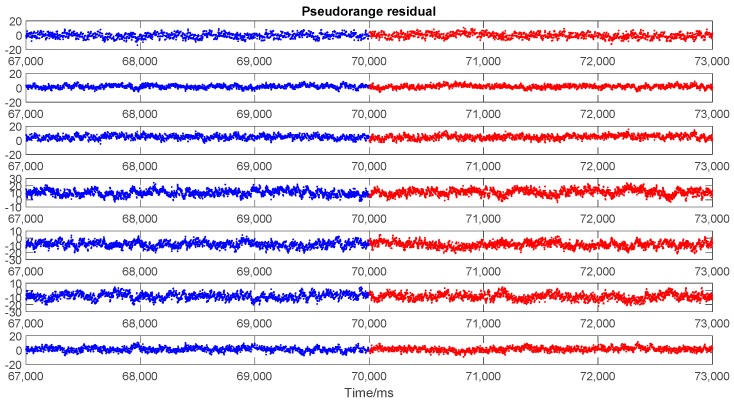
Pseudo-range residuals in every tracking channels (for 7 satellite observations). The blue and red points represent the outputs of actual and spoofing signal, respectively.

**Figure 13:**
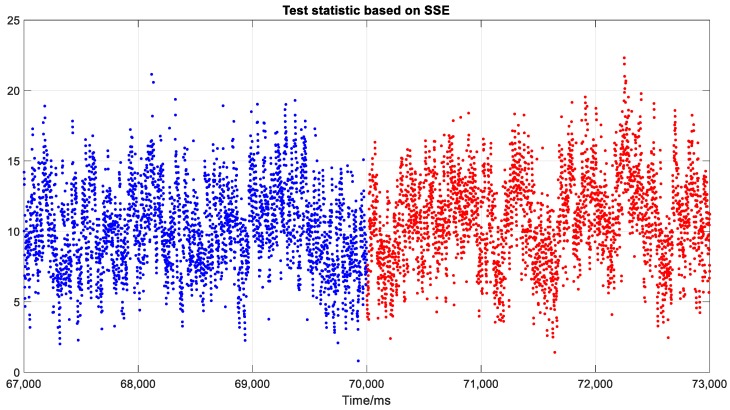
Test statistics based on squares of the residual errors (SSE). Blue and red points represent the outputs of actual and spoofing signal, respectively.

**Figure 14:**
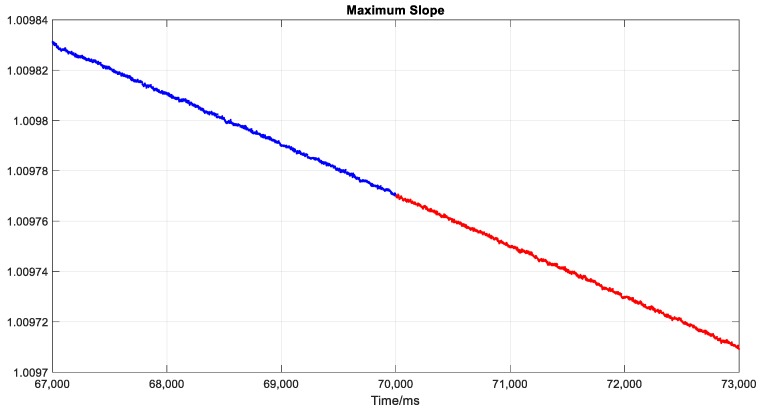
Maximum slope of geometry in receiver autonomous integrity monitoring (RAIM). Blue and red points represent the outputs of actual and spoofing signal, respectively.

Moreover, the other widely-used methods that aim to check the pseudo-range consistency to detect spoofing attack will not be effective for the proposed spoofing approach. These methods are generally applied in the positioning domain and are based on RAIM or pseudo-range residual detection. Spoofing attacks on only one or several satellites, or spoofed signals inconsistent in different channels are easily exposed to this kind of consistency detection; however, they are ineffective when all signals are spoofed. Figure 12, Figure 13 and Figure 14 show three representative parameters around the transition point for consistency checking. Figure 12 shows the pseudo-range residuals in all channels. Figure 13 is the test statistics based on sum of the squares of the residual errors (SSE). Figure 14 shows the maximum slope for the geometry in RAIM. The detailed calculation method of the above parameters can be found in [41].

As shown in Figure 12, although the residuals in different channels were different, there was no abnormal change around the transition point. The vector tracking proved its effectiveness as the LOS consistency could be guaranteed exactly for all visible satellites. Thus, spoofing detection based on checking consistency of pseudo-range residuals was incapable of detection of the spoof attack.

Test statistics and maximum slope are important parameters for classical RAIM fault detection and protection level check. The spoofing detection alarm in RAIM will be triggered only when the test statistics exceeds a threshold. As shown in Figure 13, there was no obvious change before and after the transition point, and the threshold was hard to be set in this circumstance. The maximum slope shown in Figure 14 also kept the same trend after the spoofing attack began, which verified the time consistency of the geometry matrix further. 

## 5. Discussion

In the above experimental test and performance evaluation, the spoofing generator shows superiority in signal features and observation consistency. As the actual signal component has been blocked and the spoofing signal component is closely similar to that of the actual signal, it is difficult to detect this attack based on the resulting differences of tracking channels between neighbored epochs or the snapshot consistency at the present epoch.

**Figure 15:**
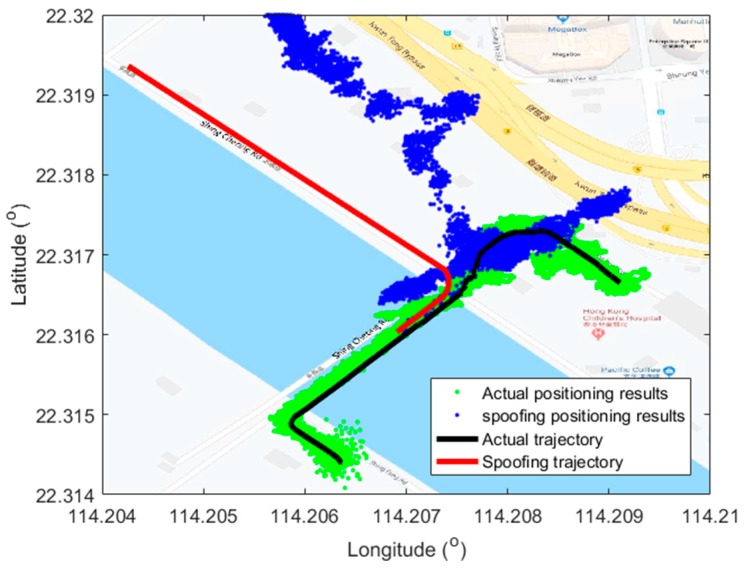
Positioning results under repeater attack. The green points, blue points, black line, and red line are positioning results under actual signal, positioning results under repeater spoofing signal, the actual trajectory, and spoofing trajectory, respectively.

**Figure 16:**
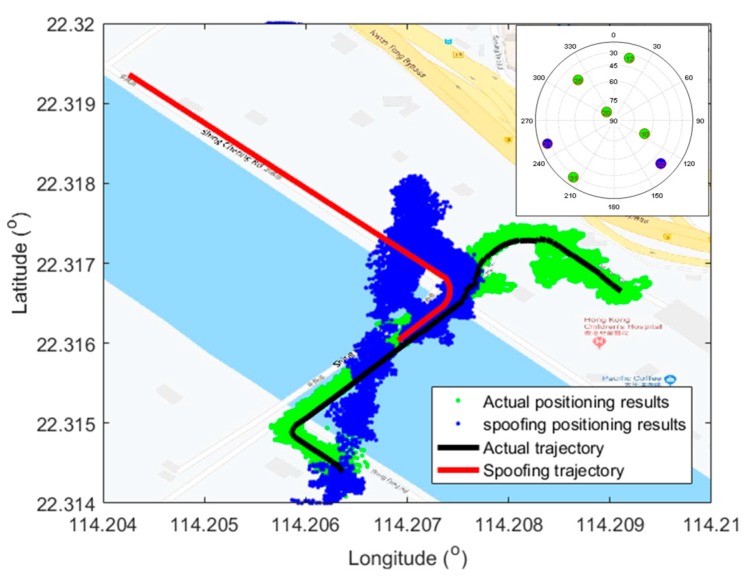
Positioning results under the scenario where PRN 21 and PRN 31 were not spoofed. The sky plot of satellites is shown in the top right corner, where green and blue numbers represent the satellites spoofed and not spoofed, respectively.

Compared to the traditional spoofing methods, another advantage of the proposed spoofing generation method is that it is trajectory driven. The superiority of vector-tracking is well utilized to covert the spoofing trajectory to the code and carrier trends of all open sky satellites. The traditional spoofing methods cannot spoof the victim receiver to the deliberate destination as planned. As shown in Figure 15, it is the attack results under a classical repeater, which is also known as meaconing. This attack recorded the actual GNSS signal and replayed after a set delay. This kind of attack is easy to be implemented and may work well in a very short time. However, the spoofing trajectory is uncertain and easy to notice due to the urban road constraints. On the other hand, once the spoofing signal does not cover whole open sky satellites perfectly, as shown in Figure 16, it also failed to guide the victim receiver along the designed trajectory.

The limitation of the proposed spoofing generator is that this kind of spoofing is based on actual signals. It needs to track the actual signal for a period of time to calculate the visible satellites, the corresponding ephemeris, the signal power, and other useful channel features. Besides, it is applicable for non-overlapped scenarios and under only GNSS available circumstances. The actual signal arriving at the victim receiver needs to be blocked to avoid the overlapped uncertainty. The information supported from other sensors or antenna is not considered in this spoofing attack scheme. What cannot be ignored is its reliance on the vector tracking receiver. In the case that vector tracking cannot guarantee its performance, the performance of the proposed spoofing attack will be compromised as well. It is believed that advanced filtering technologies [42,43] and model selection methods [44,45] will help to improve the tracking of actual signals and prediction of spoofing signals in challenging environments.

The above results are based on the assumption that the non-overlapped scenario has been created. The researchers are researching on the non-overlapped scenario implementation based on 3DMA in urban environments and will investigate methods that can rapidly detect this advanced type of spoofing in the future work.

## 6. Conclusions

A GPS spoofing generator using vector tracking-based SDR is proposed in this paper. With the help of a non-overlapped scenario, the internal nulling spoofing attack is carried out by modifying the actual signal and cancelling the actual component with the spoofing component. With the superiority of SDR vector tracking architecture, it is easy to convert the spoofing trajectory to the corresponding code and carrier. The modified signal still maintains the actual amplitude, satellite ephemeris, and other important signal features. The test results show that the spoofing attack can work effectively, and the receiver was misled to the spoofed trajectory successfully. The spoofing detection methods in track channel or positioning domain have difficulty detecting this spoofing as the spoofing signal keeps high consistency in tracking features and observation pseudo-ranges. There is no abnormal change in the tracking results or positioning solutions. The threat of this spoofing mode to autonomous vehicles is hazardous once all the visible GPS satellites are spoofed.

As it is undeniable that there is an actual and urgent need to research on spoofing generators, the above spoofing generator, implemented based on an open source SDR with a mature vector tracking architecture, will help the research on spoofing defenses in the future.
